# Supplementary material for: Sulforaphane Is Protective against Warm Ischemia/Reperfusion Injury and Partial Hepatectomy in Rats
Source: Int J Mol Sci. 2024 Jan 1;25(1):579. doi: 10.3390/ijms25010579 (PMC10778753; doi:10.3390/ijms25010579)
Supplement: Supplementary file 1 [file ijms-25-00579-s001.zip › ijms-2774544-supplementary.pdf]

Supplementary Table S1 Primers used for RT-PCR to detect mRNA

	Sequence (5′ → 3′)	Direction	Length (bp)
CXCL1	GCCAAGCCACAGGGGCGCCCGT	Forward	231
	ACTTGGGGACACCCTTTAGCATC	Reverse	
TNF-α	TCCCAACAAGGAGGAGAAGTTCC	Forward	275
	GGCAGCCTTGTCCCTTGAAGAGA	Reverse	
IL-10	GCAGGACTTTAAGGGTTACTTGG	Forward	245
	CCTTTGTCTTGGAGCTTATTAAA	Reverse	
MCL1	CTGGGGCAGGATTGTGACTC	Forward	169
	CACAAACCCATCCCAGCCTCTTTG	Reverse	
HGF	GCTACACTGGATTGATCAACGC	Forward	169
	CCATAATCTCCCTCACAAGGTC	Reverse	
IL-6	GAGAAAAGAGTTGTGCAATGGCA	Forward	137
	ATAGGCAAATTTCCTGGTTATATCC	Reverse	
IL-1β	TCTTTGAAGAAGAGCCCGTCCTC	Forward	321
	GGATCCACACTCTCCAGCTGCA	Reverse	
EF	TCTGGTTGGAATGGTGACAACATGC	Forward	335
	CCAGGAAGAGCTTCACTCAAAGCTT	Reverse	

RT-PCR reverse transcription-polymerase chain reaction, *bp* base pairs, *TNF-α* tumor necrosis factor-alpha, *CXCL1* C-X-C motif chemokine ligand 1, *IL* interleukin, *MCL1* myeloid cell leukemia 1, *HGF* hepatocyte growth factor, *EF* elongation factor 1α

Supplementary Table S2 Histological index in the liver of HIRI+PH-treated rats

	Average ± SD	P value No treatment vs SFN	P value HIRI+PH 3 h vs 6 h
Suzuki score			
3 h			
HIRI+PH	4.0 ± 0.0		
HIRI+PH and SFN	1.0 ± 0.0	0.044	
6 h			
HIRI+PH	9.3 ± 1.2		0.002
HIRI+PH and SFN	3.5 ± 1.7	<0.001	
TUNEL-positive cells/mm^2			
3 h			
HIRI+PH	0.5 ± 0.8		
HIRI+PH and SFN	0.0 ± 0.0	1.000	
6 h			
HIRI+PH	169.0 ± 45.9		<0.001
HIRI+PH and SFN	66.6 ± 36.1	0.002	
MPO-positive cells/mm^2			
3 h			
HIRI+PH	17.8 ± 4.1		
HIRI+PH and SFN	21.7 ± 4.8	0.992	
6 h			
HIRI+PH	79.2 ± 29.5		0.006
HIRI+PH and SFN	31.6 ± 6.3	0.018	

No changes in pathological findings or no positive staining were clearly detected in the livers of control (0 h, naïve rats).

HIRI+PH hepatic ischemia/reperfusion injury and partial hepatectomy, *SFN* sulforaphane, *TUNEL* terminal deoxynucleotidyl transferase-mediated deoxyuridine nick-end labeling, *MPO* myeloperoxidase.

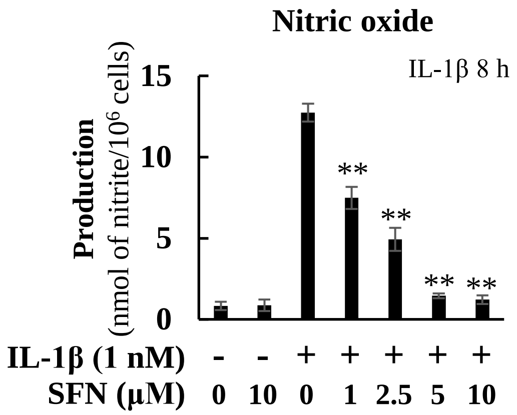

Supplementary figure S1. Dose-dependent effects of sulforaphane (SFN) on nitric oxide production in interleukin 1β (IL-1β)-stimulated hepatocytes. Cells were treated with IL-1β (1 nM) in the presence or absence of SFN (1 to 10 μM) for 8 h. The medium concentration of nitrite, a metabolite of nitric oxide, was determined by the Griess method. **P < 0.01 versus IL-1β alone. The values in the bar graphs represent the mean ± standard deviation (SD; *n* = 3 experiments).
